# Characterization of *Lactobacillus spp.* isolated from layer hens as probiotic candidates

**DOI:** 10.1186/s12917-025-04847-0

**Published:** 2025-07-02

**Authors:** Jiawei Zhang, Andrea R. McWhorter, Samiullah Khan, Nicky-Lee Willson, Kapil K. Chousalkar

**Affiliations:** School of Animal and Veterinary Sciences, The University of Adelaide, Roseworthy, SA 5371 Australia

**Keywords:** Layer hen, *Lactobacillus*, Gut health, Probiotics, *Salmonella*

## Abstract

**Background:**

Egg production and performance in commercial layer flocks can be impacted by gastrointestinal health. Probiotics are considered potential alternatives to antibiotics in poultry, and have been proven to have positive impacts on gut health. The objective of this study was to isolate and characterise *Lactobacillus* strains isolated from high production layer flocks for probiotic properties.

**Results:**

Eleven different *Lactobacillus* (*L.*) species were isolated from faecal samples collected from layer hens in late lay. Species included *L. agilis*, *L. crispatus*,* L. gallinarum*,* L. ingluviei*,* L. johnsonii*,* L. kitasatonis*,* L. mucosae*, *L. oris*, *L. reuteri*, *L. saerimneri* and *L. salivarius*. *Enterococcus cecorum* and *Enterococcus faecium* as well as *Streptococcus alactolyticus* were also isolated. Phenotypic properties of the eleven *Lactobacillus* species were then evaluated to assess their probiotic potential. Bacterial culturability at low pH, under high concentrations of bile salt and sodium chloride, as well as in simulated gastric juice were characterised for each species. Auto- and co-aggregation capacity, adhesion to the intestinal epithelium as well as inhibition of *Salmonella* Typhimurium were also evaluated. All the *Lactobacillus* spp. exhibited high culturability in sodium chloride at different temperatures, and exhibited strong adhesion to chicken epithelial cells. *L. reuteri*,* L. salivarius*,* L. saerimneri*, and *L. muc*o*sae* displayed higher culturability in low pH conditions, and *L. johnsonii* displayed higher culturability in high concentration of bile salt. Only *L. crispatus* was culturable in simulated gastric juice after 24 h incubation. *L. kitasatonis* exhibited strong auto-aggregation and co-aggregation with wild-type *Salmonella* Typhimurium. All *Lactobacillus* species exhibited strong inhibition activity against *Salmonella* Typhimurium.

**Conclusions:**

The *Lactobacillus* species isolated in this study exhibited probiotic properties. Further in vivo experiments are required to evaluate their capacity to colonise and persist in the host as well as evaluate their potential effects on the gut microbiota and overall health parameters of layer hens.

## Introduction

Enhancing layer hen productivity is important for the economic sustainability of the egg industry. Egg producers invest in several strategies to maintain bird health including nutritional management, husbandry practices, biosecurity measures, vaccination, as well as strengthening the gut microbiota [1]. A healthy gut microbiota plays important roles in nutrient absorption, immune function, and overall gut health that directly influences performance, egg production, and quality [2–5]. Strategies that promote a balanced and diverse gut microbiota can enhance the gut health of birds, reduce the risk of disease, and contribute to egg production. The use of probiotics is a common strategy employed to shape and maintain a healthy gut microbiota.

The Food and Agriculture Organization of the United Nations (FAO) and World Health Organisation define probiotics as “live microorganisms that, when administered in adequate amounts, confer a health benefit on the host” [6, 7]. Use of probiotics has been linked with improved gastrointestinal integrity and function, digestion, as well as immunity [8]. Probiotic supplements developed for poultry most frequently include one or more of the bacterial genera *Bacillus*, *Bifidobacterium*, *Enterococcus*, *Lactobacillus*, and *Streptococcus* but also sometimes include yeast or other fungal species [5].

Over the past several decades, there has been considerable research investigating the potential health benefits of different *Lactobacillus* strains. Members of *Lactobacillaceae* family are Gram positive, non-spore forming, facultative or strict anaerobic bacteria that are either homofermentative or heterofermentative and the primary product of fermentation is lactate [9]. This lactic acid producing bacterial family is comprised of 26 genera containing over 260 species [9]. They are dominant members of the gut microbial community and contribute to the digestion of complex carbohydrates [9]. Short-chain fatty acids (SCFAs) including propionate, butyrate, and lactate are produce by *Lactobacillus* spp. during fermentation of nondigestible carbohydrates [10]. These SCFAs contribute to intestinal barrier function and reduce the pH of gut contents which inhibits the colonisation of the gastrointestinal tract by bacterial pathogens [11]. It has been shown that SCFAs can also positively influence feed efficiency [12].

The poultry gut microbiota is a highly diverse and dynamic ecosystem that can be influenced by several factors including breed, housing environment, and diet [5]. As a bird ages, changes in the gastrointestinal physiology also contribute to temporal changes in the composition of the gut microbiota [13–15]. Studies focussed on broiler birds commonly isolate *Lactobacillus reuteri*, *Lactobacillus johnsonii*, *Lactobacillus acidophilus*, *Lactobacillus crispatus*, *Lactobacillus salivarius*, *Lactobacillus fermentum*,* Lactobacillus vaginalis*,* Lactobacillus ingluvei*,* Lactobacillus saerimneri*, and *Lactobacillus aviaries* from freshly collected faecal material [16–18]. *Pediococcus acidilactici*, *Pediococcus pentosaceus*, and *Enterococcus faecium* are also often isolated from broiler faeces [19]. Similar *Lactobacillus* species have been isolated from layer hens [20, 21].

Candidate probiotic bacterial species need to exhibit genotypic and phenotypic stability, tolerance to acid and bile, the capacity to adhere the intestinal epithelium, and inhibition of gut pathogens [22]. For example, strains of *Lactobacillus crispatus* have been identified as a potential probiotic because they have been found to exhibit strong adhesive capacity to intestinal epithelium [23] and are tolerant to high concentrations of bile [24]. *Lactobacillus reuteri* strains have also exhibited acid and bile tolerance [25] and possess competitive exclusion properties against pathogenic *Escherichia coli* and *Salmonella* spp [26]. In layer hens, *Lactobacillus reuteri* has exhibited the capacity to competitively exclude *Salmonella* Enteritidis [20].

The gut microbiota of high-producing layer hens has been shown to differ from that of low-producing hens, with a higher abundance of *Lactobacillus* species in high-production birds [27]. This suggests that *Lactobacillus* species isolated from high-production layer hens could exhibit probiotic characteristics that, when administered, could enhance gut health and overall egg production efficiency. The overarching aim of this study was to characterise promising probiotic candidates that can contribute to layer hen gut health. To exert their beneficial effects, probiotic bacterial strains must possess specific characteristics [22]. In this study, the in vitro phenotypic attributes of *Lactobacillus* strains isolated from layer hen faeces were investigated to determine their probiotic potential. The ability of different *Lactobacillus* species to survive at low pH, in bile salt, and sodium chloride (NaCl), simulated gastric juice was investigated. Additionally, bacterial thermostability as well as characterisation of adherence to the epithelium of ileal and caecal tissue were evaluated. The ability of each *Lactobacillus* species to inhibit *Salmonella* Typhimurium was also investigated.

## Methods

### Isolation of *Lactobacillus* spp from layer hen faeces

Fresh faecal samples were collected from 13 South Australian commercial layer hen flocks all aged 60 weeks or older. All flocks had high production based on industry standards. Permission to collect faeces was obtained from egg producers and collection procedures were approved by the University of Adelaide Animal Ethics Committee, approval number S-2023-030. From each flock, ten fecal samples were collected from the shed infrastructure. Samples were maintained on an ice pack until processed on the day of collection for *Lactobacillus* spp isolation. Prior to isolation one gram of each sample were combined and thoroughly mixed in a separate sterile 250 mL sample container (Technoplas, Australia).

*Lactobacillus spp.* isolation was performed by suspending 1 g of fecal sample into 9 mL de Man, Rogosa, Sharpe (MRS) broth (BD Difco, Edwards Group, Australia) and vortexed for 30 s. Serial 10-fold dilutions were prepared by adding 100 µL of the diluted fecal sample into 900 µL of 0.9% saline. One hundred µL of each dilution was spread-plated onto an MRS agar plate and incubated anaerobically at 37 °C for 48 h in an Oxoid compact plastic pouch (ThermoFisher Scientific, Australia) containing an AnaeroGen™ 2.5 L pack (ThermoFisher Scientific, Australia). Based on morphology, unique individual colonies were selected and sub-cultured onto fresh MRS agar plates. This process was repeated until pure cultures were obtained. A culture was defined as pure once colonies of uniform appearance were observed on each plate. This was achieved within two passages for each isolate.

Isolates were identified using matrix-assisted laser desorption/ionisation time of flight mass spectrometry (MALDI-TOF) (Bruker Microflex LT) (Roseworthy Veterinary Hospital, Department of Pathology, The University of Adelaide). Three colonies from each pure culture were used for MALDI-TOF identification. To prepare stocks, a single colony was added to 9 mL of MRS broth in a 15 mL conical tube (Nunc, ThermoFisher Scientific) and incubated at 37 °C under anaerobic conditions (maintained in a Oxoid AnaeroJar 2.5 L with an AnaeroGen sachet) in a shaking incubator (Ratek, Adelab Scientific, Australia) at 150 rpm for 24 h. Three hundred µL of each bacterial culture was mixed with an equal volume of 100% glycerol in a 2 mL microcentrifuge tube (Eppendorf, Australia), and stored at -80 °C.

### Evaluation of haemolytic activity

*Lactobacillus* isolates were streaked onto Columbia agar containing 5% sheep blood (Oxoid, Australia). Plates were incubated at 37 °C anaerobically. After 48 h incubation, plates were evaluated for the presence of $\:\alpha\:$ (partial), $\:\beta\:$ (complete), or $\:\gamma\:$ (non-haemolysis) haemolysins.

### Experimental design and preparation of bacterial suspensions for in vitro tests

The number of individual isolates for *Lactobacillus* species varied. To assess tolerance to acidic environments, bile salts, osmotic stress, and temperature stress, each isolate was streaked from frozen stock onto two separate MRS agar plates and incubated anaerobically at 37 °C overnight. A single colony of each isolate was added to 10 mL MRS broth previously added to a 15 mL conical tube and incubated anaerobically in AnaeroJars with AnaeroGen sachets at 37 °C for 24 h with shaking (150 rpm). Cultures were adjusted to an optical density (OD) at 600 nm of 0.2, equivalent to 10^8^ CFU/ml [28]. Four biological replicates for each isolate were included in each experiment.

In tolerance experiments, no significant difference was observed between isolates of the same species. For auto-aggregation, co-aggregation, intestinal adherence, and *Salmonella* inhibition experiments, a single isolate was used for each experiment. Each experiment included two technical replicates and were repeated three times.

### Tolerance of *Lactobacillus* species to acidic environments

MRS broth was adjusted to pH 2, 3, and 4 using 1 M hydrochloric acid (HCl). For these experiments, 900 µL of pH-adjusted MRS broth was added to 2 mL dilution tubes (SSI Bio, USA) that had been added to a 96 well dilution tube rack. One hundred µL of each bacterial suspension was then added separately to each tube and covered with the plate lid to prevent evaporation. The plate was incubated anaerobically at 37 °C for 24 h with shaking (150 rpm). At each time point, serial 10-fold dilutions were prepared for each treatment. Ten µL of each dilution were drop-plated onto MRS plates. All agar plates were incubated anaerobically at 37 °C for 24 h. Bacteria were enumerated and data are presented as log_10_ CFU/mL over time.

### Culturability of *Lactobacillus spp.* in simulated gastric juice

Simulated gastric juice was prepared as previously described by [29] with modifications. For 500 mL of gastric juice, 1.75 g glucose, 1.025 g NaCl, 0.30 g KH_2_PO_4_, 0.505 g CaCl_2_, and 0.185 g KCl were dissolved into 500 mL of reverse osmosis water and adjusted to a pH of 3.0 with 1 M HCl and autoclaved to sterilise. Prior to experiments, a stock solution of pepsin (Sigma, Australia) was added to the stimulated gastric juice to a final concentration of 6.65 mg in 500 mL [29]. Prior to experiments, bacterial suspensions were prepared to match a MacFarland 0.5 standard. One hundred µL of bacterial suspension were inoculated into dilution tubes containing 900 µL of simulated gastric juice. Samples were then incubated anaerobically at 37 °C for 24 h with shaking (150 rpm). The load of culturable bacteria was evaluated at 0, 4 and 24 h of incubation. Serial dilutions and plating were performed as above.

### Bile salt tolerance

Sterile MRS broth containing 1, 3, or 5% bile salts (Sigma-Aldrich, Australia) was prepared. Bacterial suspensions were prepared to match a MacFarland 0.5 standard. One hundred µL of each bacterial suspension was added to a separate tube containing 900 µL of MRS broth with either 1, 3, or 5% bile salts and incubated anaerobically at 37 °C for 24 h with shaking (150 rpm). Bacterial culturability was evaluated at 0-, 4- and 24-hours post-inoculation. Serial 10-fold dilutions were prepared for each sample and drop-plated onto MRS agar plates. Plates were incubated anaerobically at 37 °C for 24 h. Bacteria were enumerated and data are presented as log_10_ CFU/mL.

### Tolerance of *Lactobacillus spp.* to osmotic stress

MRS broth was prepared with increasing concentrations (1%, 3%, 5%, and 7%) of NaCl and aliquoted into 1 mL dilution tubes. One hundred µL of each bacterial suspension were added to 900 µL of MRS containing NaCl. Culturable bacteria were enumerated at 0-, 4- and 24-hours post-inoculation to characterise the osmotic tolerance of the *Lactobacillus* isolates. As described above, serial 10-fold dilutions were prepared for each treatment and drop-plated onto MRS agar plates. Plates were incubated anaerobically at 37 °C for 24 h. Bacteria were enumerated and data are presented as log_10_ CFU/mL.

### Culturability of *Lactobacillus* spp at different temperatures

*Lactobacillus* cultures were prepared as described above. Four mL of each isolate suspensions were then incubated at 4 °C, 25 °C, and 37 °C anaerobically for 48 h. The culturability of each isolate was measured at 4, 24, and 48 h. At each timepoint, serial 10-fold dilutions were prepared and 10 µL of each treatment sample were drop-plated onto MRS agar plates. Plates were incubated anaerobically at 37 °C for 24 h. Bacteria were enumerated and data presented as log_10_ CFU/mL.

### Autoaggregation of *Lactobacillus* species

Stationary phase *Lactobacillus* cultures were collected by centrifugation for 10 min at 4,500 *× g*. Bacterial pellets were washed once and resuspended in phosphate buffered saline (PBS). The bacterial concentration was adjusted to approximately 10^8^ CFU/mL. Samples were held at room temperature for two hours without disruption of the microbial suspension. Optical density 600 nm was measured using a spectrophotometer (ClarioStar, BMG Labtech, Australia). The autoaggregation coefficient (AC) was calculated as per previously published methods [30, 31]. OD_i_ is the initial OD 600 nm at time 0 and OD_f_ is the optical density at time 2 h.

$$\:\text{E}\text{q}\text{u}\text{a}\text{t}\text{i}\text{o}\text{n}\:1:\:\text{A}\text{C}/\text{C}\text{A}=\left[\frac{{\text{O}\text{D}}_{\text{i}}-{\text{O}\text{D}}_{\text{f}}}{{\text{O}\text{D}}_{\text{i}}}\right]\times\:100$$

### Co-aggregation with *Salmonella* Typhimurium

A *Salmonella* Typhimurium phage type 9 isolate previously cultured from a commercial layer hen environment was used to test co-aggregation (CA) with each of the *Lactobacillus* species isolated in this study. Prior to experiments, the ST isolate was resuscitated from a -80 °C stock by streak plating onto a nutrient agar plate (Oxoid, Australia) and incubated at 37 °C for 24 h aerobically. Stationary phase cultures of both the *Salmonella* Typhimurium and all *Lactobacillus* species were prepared as above. Bacterial pellets were washed once and resuspended in PBS and adjusted to a bacterial concentration of log 8 CFU/mL. The co-aggregation test was conducted by mixing equal volumes of the *Lactobacillus* and *Salmonella* Typhimurium suspensions into each well of a deep 96-well plate. OD 600 nm was measured at time 0 and again after two hours of incubation. Percent co-aggregation was calculated as shown in Eq. 1.

### Adhesion of *Lactobacillus spp.* to chicken gut epithelium

The adhesion capacity of the *Lactobacillus* isolates was evaluated using ileal and caecal tissue obtained from one- and two-week-old layer chicks. Tissues were scavenged from a separate study conducted with approval from the University of Adelaide’s Animal Ethics Committee (approval number S-2023-030). For the experiments, day old HyLine Brown layer hen chicks were obtained from a commercial hatchery. Chicks were housed in pens lined with thick brown paper and were provided feed and water *ad libitum*. At one and two weeks of age, chicks were humanely killed by cervical dislocation. All experiments were conducted under the Australian Code for Care and Use of Animals for Scientific Purposes.

Upon collection, sections of the ileum and caeca were maintained on ice in phosphate-buffered saline (PBS). Subsequently, tissues were thoroughly washed in 0.9% saline solution to remove the intestinal contents. Intestinal sections were cut open longitudinally with a dissecting scissors and opened with the epithelial side facing upward. A 1 cm biopsy punch (Kai Medical, Japan) was used to excise circular size samples from the tissue segments.

Each punch was placed in an individual well of a 48-well cell culture plate (ThermoFisher, Australia). Samples were then inoculated with 200 µL of a stationary phase *Lactobacillus* culture. Samples were incubated anaerobically in a shaking incubator at 100 rpm for 2 h. Following this incubation period, the tissues were washed three times with 0.9% saline to remove bacteria that had not adhered to the epithelium. Punches were then homogenised, and serial 10-fold dilutions were prepared. Dilutions were drop plated (10 µL volume) onto MRS plates. Plates were incubated anaerobically at 37 °C for 24 h. Bacteria were enumerated and data were presented as CFU/gram of tissue.

### Inhibition activity against *Salmonella* Typhimurium during co-inhibition with *Lactobacillus spp.*

Stationary phase cultures of each *Lactobacillus* strain were prepared prior to experiments. Following two washes with PBS, the bacterial pellet was resuspended in Mueller Hinton broth (Oxoid, Australia) and adjusted to an OD600 nm of 0.2 (10^8^ CFU/mL). The *Salmonella* Typhimurium inoculum was prepared as for the co-aggregation experiments. Subsequently, 2 mL of each *Salmonella* Typhimurium and *Lactobacillus* suspension were mixed in 15 mL tubes. A *Salmonella* Typhimurium and *Lactobacillus* only cultures were included as controls. Samples were incubated at 37 °C, and *Salmonella* Typhimurium and *Lactobacillus* loads were characterised at 2, 4 and 24 h post incubation. Serial 10-fold dilutions were prepared at each time point and 10 µL from each treatment was drop plated onto both MRS and XLD plates.

### Data analysis

Statistical analyses were performed using GraphPad Prism version 10.4.1. A D’Agostino and Pearson test was conducted to test for normal distribution of data. Where appropriate, non-parametric tests were used for non-normally distributed data. In tolerance experiments, no significant difference was observed between isolates of the same species. All isolates were combined and analyses were performed with species as a variable. Because of the unequal number of isolates used in the acid tolerance, bile salt, osmotic stress, and temperature experiments, a Friedman’s test was used evaluate the effects of different environmental conditions at different timepoints. *Lactobacillus* inhibition of *Salmonella* Typhimurium over time was also analysed using a two-way ANOVA. Due to non-normal distribution, a Kruskal-Wallis with a post hoc Dunn’s multiple comparison test was used to evaluate statistical significance for epithelial adhesion, co-aggregation, and auto-aggregation activity. In all cases, a *p*-value of < 0.05 was considered statistically significant.

## Results

### *Lactobacillus* isolation from layer hen faeces

Eleven different *Lactobacillus* (*L.*) species were isolated from layer hen faecal samples including *L. agilis*, *L. crispatus*,* L. gallinarum*,* L. ingluviei*,* L. johnsonii*,* L. kitasatonis*,* L. mucosae*, *L. oris*, *L. reuteri*, *L. saerimneri* and *L. salivarius*. *Enterococcus cecorum* and *Enterococcus faecium* as well as *Streptococcus alactolyticus* were also isolated. Among the *Lactobacillus* isolates, *L. salivarius* was the most isolated species representing 27.9% of the total. In contrast, *L. ingluviei*, *L. mucosae*, and *L. oris* were least frequently isolated each representing only 0.8%. Five isolates of each of *L. agilis*,* L. crispatus*,* L. gallinarum*, *L. johnsonii*, *L. kitasatonis*,* L. reuteri*, and *L. salivarius*, three of *L. saerimneri*, and two of *L*. *ingluviei*, and *L*. *mucosae*, and *L. oris* were used for downstream characterisation. None of the *Lactobacillus* isolates were haemolytic when grown on 5% sheep blood agar.

### *Lactobacillus* strain tolerance to acidic conditions

Potential probiotic bacterial species must be able to survive in the host gastrointestinal system, which presents numerous environmental challenges, such as low pH. The pH of the chicken gastrointestinal tract varies between each section. The proventriculous and gizzard generally have the lowest pH ranging between 2.6 and 4.2 [5, 32]. In the present study, the 11 *Lactobacillus* isolates were evaluated for their sensitivity to low pH (pH 2, 3, and 4) over 24 h. Bacteria were suspended in MRS broth acidified with 1 M HCl and culturability was evaluated at 0, 4, and 24 h. Data are presented as the mean log_10_ CFU/mL $\:\pm\:$ standard error.

**Fig. 1:**
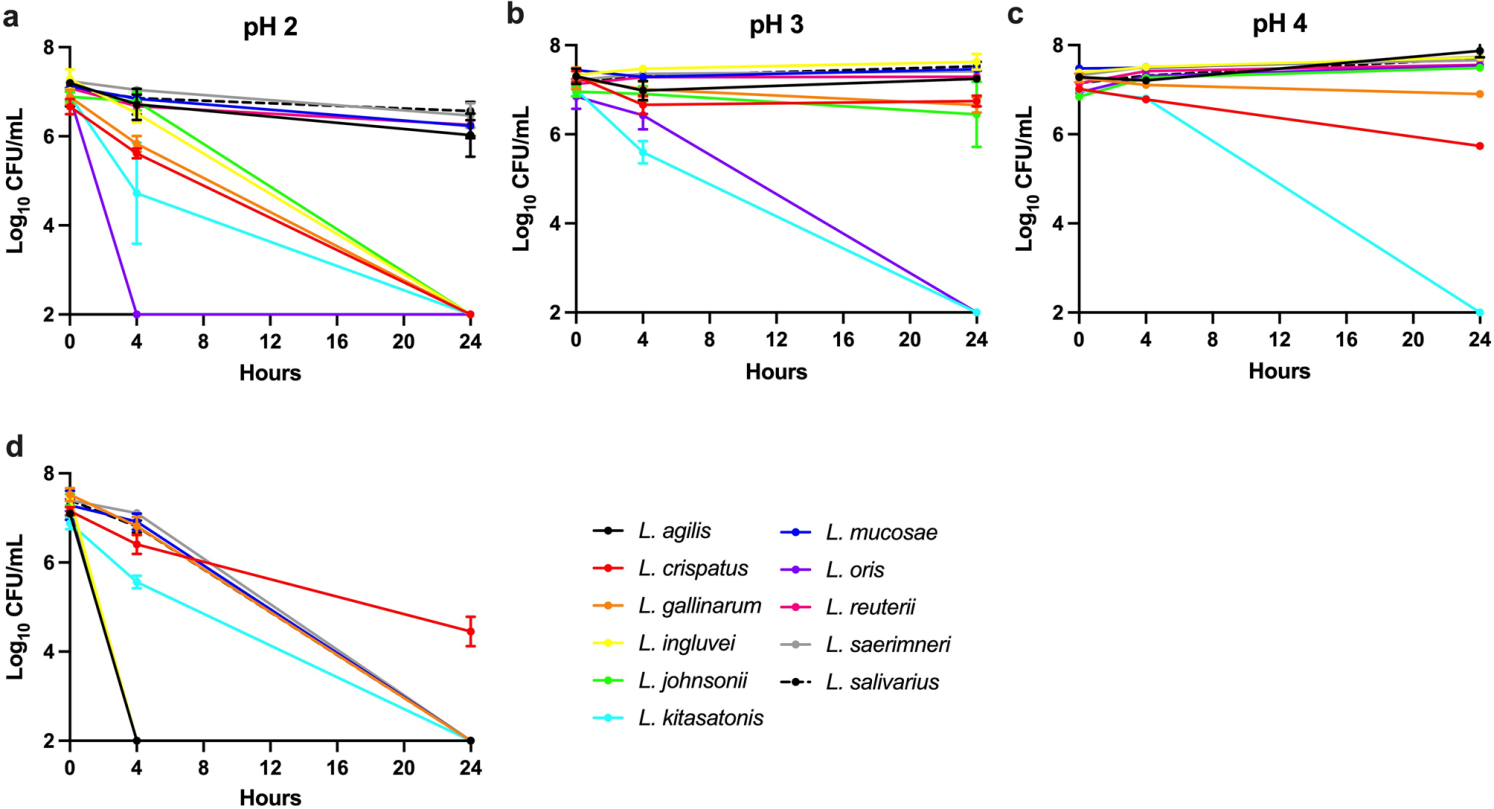
Tolerance of *Lactobacillus* to low pH. Culturability of each of the 11 *Lactobacillus* species was evaluated in MRS broth acidified to pH 2 (**a**), pH 3 (**b**), and pH 4 (**c**). Bacteria were also incubated in simulated gastric juice (pH 3) (**d**) to evaluate the effect of low pH in the presence of pepsin

At pH 2, *L. oris* isolates were the most sensitive and after 4 h incubation no culturable bacteria were observed (Fig. 1a). After 4 h, *L. kitasatonis*, *L. crispatus*, and *L. gallinarum* all exhibited a significant decrease (p $\:\le\:$ 0.001) in their culturability and after 24 h, were no longer culturable. The culturability of *L. ingluviei* and *L. johnsonii* was not affected after 4 h but they were no longer culturable at 24 h. *L. agilis*, *L. mucosae*, *L. reuteri*, *L. salivarius*, and *L. saerimneri* all exhibited tolerance to pH 2, remaining culturable over the 24 h experiment. These five *Lactobacillus* isolates: however, did exhibit a significant (*p* < 0.0001) decline in culturability over time (Fig. 1a). Percent reductions for these species ranged between 6.6 and 16.2%.

*L. kitasatonis* and *L. oris* exhibited the highest acid sensitivity at pH 3 (Fig. 1b). A significant decrease (p $\:\le\:$ 0.001) in bacterial load was observed for both isolates after 4 h. After 24 h, neither *L. kitasatonis* nor *L. oris* were culturable. *L. crispatus*, *L. gallinarum*, *L. johnsonii* all exhibited a decrease in culturability after 4 h incubation at pH 3 but the differences were not statistically significant. Culturability of these three strains remained stable for the remainder of the experiment. All other *Lactobacillus* isolates were tolerant to pH 3 and remained culturable with no changes in bacterial load observed over time (Fig. 1b).

At pH 4, *L. kitasatonis* was the most sensitive strain. Culturability of this strain decreased after 4 h, and at 24 h, it was not culturable (Fig. 1c). *L. crispatus* and *L. gallinarum* exhibited a slow decline over the course of the experiment with percent reductions of 18.4% and 4.8% respectively. All other isolates were tolerant to a pH 4 environment and exhibited growth with a mean percent increase of 10.2%.

Bacteria were also incubated in simulated gastric juice (pH 3) to evaluate tolerance to low pH in the presence of pepsin, a proteolytic enzyme. The load of culturable bacteria decreased significantly for all *Lactobacillus* isolates (*p* < 0.0001) (Fig. 1d). *L. agilis*, *L.ingluviei*, and *L. johnsonii* were the most sensitive and were not culturable after 4 h. All other *Lactobacillus* strains exhibited continued decrease in culturability over the 24 h experimental duration. *L. crispatus* was the only isolate that remained culturable over the entire experimental duration, exhibiting a 37.3% decrease in culturability.

### Tolerance of *Lactobacillus* species to bile salts and osmotic stress

**Fig. 2:**
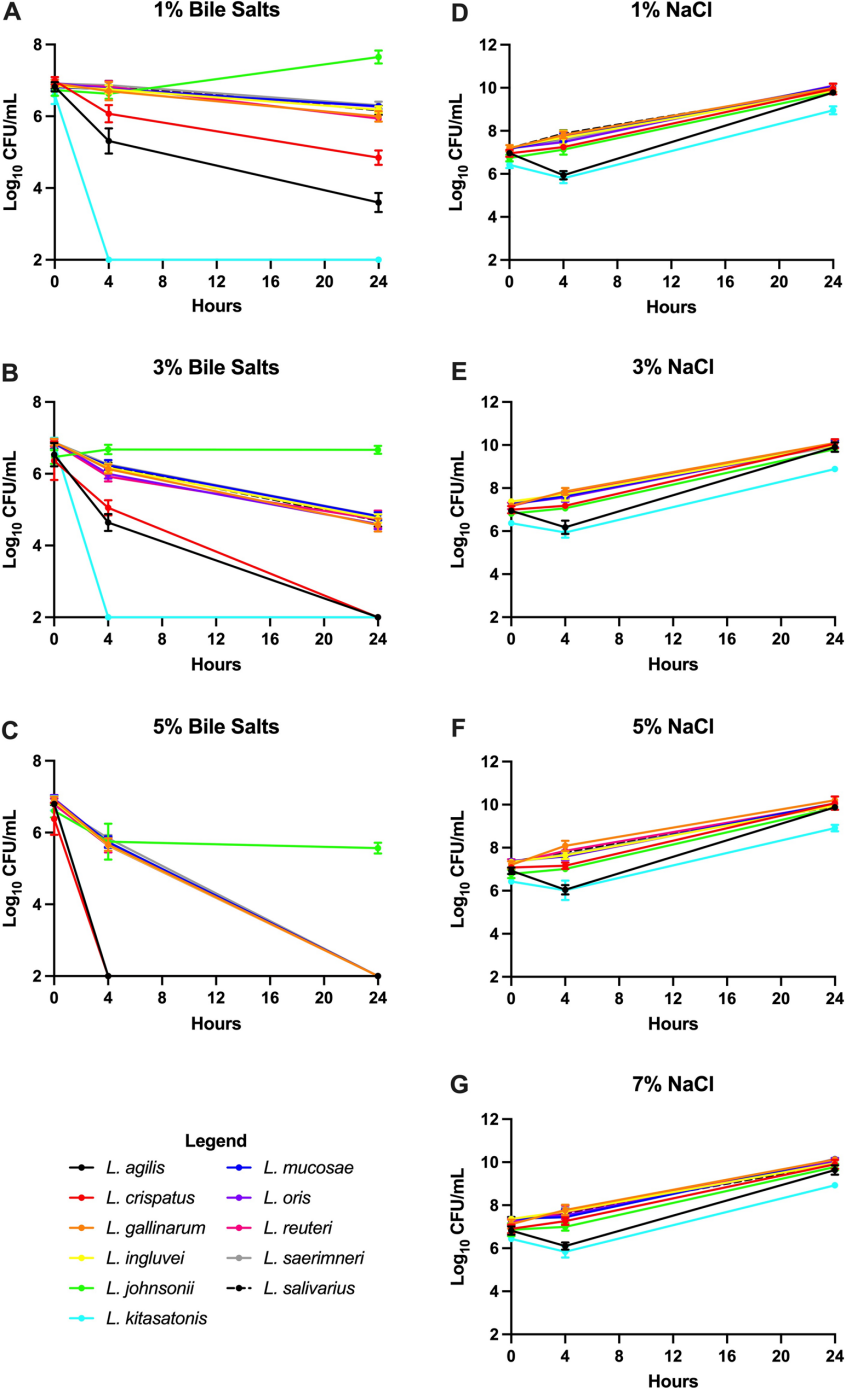
Sensitivity of *Lactobacillus* species to increasing concentrations of bile salt and NaCl. The culturability of the 11 *Lactobacillus* species isolated in this study were evaluated in the present of 1% (**a**), 3% (**b**), and 5% bile salts (**c**) as well as 1% (**d**), 3% (**e**), 5% (**f**), and 7% NaCl (**g**)

Resistance to bile salts is also an important characteristic of a probiotic strain, as it not only enhances the survivability of bacteria in the small intestine [33] but also contributes to tolerance of other stressors [34]. Tolerance to increasing concentrations of bile salt was investigated for each of the 11 layer hen associated *Lactobacillus* species (Fig. 2a, b, c). *L. kitasatonis* exhibited the highest sensitivity to bile salts; no viable bacteria were observed after 4 h incubation at all bile salt concentrations tested (Fig. 2a, b, c).

While more tolerant than *L. kitasatonis*, *L. agilis* and *L. crispatus* also exhibited sensitivity to bile salts (Fig. 2a, b, c). Both *Lactobacillus* species exhibited significant reduction (p $\:\le\:$ 0.01) in culturable bacteria after 24 h incubation in 1% bile salts. After 4 h incubation in 3% bile salts, *L. agilis* and *L. crispatus* exhibited a 1.87 and 0.85 log reduction in culturable bacteria, respectively, and by 24 h were no longer culturable. Neither *L. agilis* nor *L. crispatus* were culturable after 4 h incubation in 5% bile salts.

*L. gallinarum*, *L. ingluvei*, *L. mucosae*, *L. oris*, *L. reuteri*, *L. saerimneri* and *L. salivarius* exhibited moderate tolerance to bile salts (Fig. 2a, b, c). After 24 h incubation in 1% bile salts, these *Lactobacillus* species all remained culturable but exhibited a significant reduction (*p* < 0.05) in load with an average log reduction of 1.1. In 3% bile salts, these seven *Lactobacillus* species also remained culturable for 24 h but exhibited a mean log reduction of 2.3. These strains were highly sensitive to 5% bile salts and were no longer culturable after 4 h of incubation.

*L. johnsonii* was the most tolerant to increasing bile salt concentration (Fig. 2a, b, c). During incubation in 1% bile salt, a significant increase (*p* < 0.01) in bacterial load was observed for *L. johnsonii* over 24 h. No significant change in bacterial load was observed for *L. johnsonii* when incubated in 3% bile salts. After 4 h incubation in 5% bile salts, L. *johnsonii* exhibited a 0.83 log reduction and the load remained constant over the remainder of the experiment.

The resting baseline osmolarity of the gastrointestinal tract lumen is equivalent to approximately 0.3 M NaCl [35] but this can fluctuate significantly during feeding and digestion. Thus, tolerance to high salt concentrations is another important property of potential probiotic bacterial strains. To evaluate tolerance to osmotic stress, the *Lactobacillus* species isolated in this study were cultured in increasing NaCl concentrations of 1% (0.17 M), 3% (0.51 M), 5% (0.85 M), and 7% (1.19 M). Four hours after exposure, the culturability of both *L. kitasatonis* and *L. agilis* increased significantly (*p* < 0.01) at all NaCl concentrations (Fig. 2d, e, f, g). After 24 h, both *L. kitasatonis* and *L. agilis* exhibited an overall increase in load. Over the 24 h experiment, however, the load of all *Lactobacillus* strains increased an average of 3 logs.

### Thermal tolerance of *Lactobacillus* isolates

**Fig. 3:**
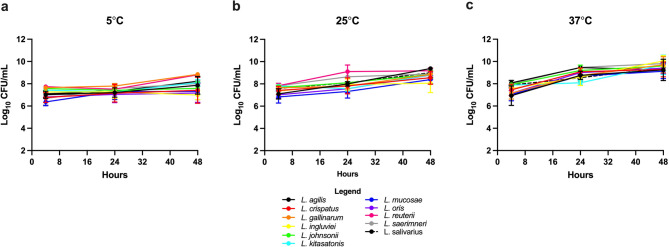
Thermostability of *Lactobacillus* species at different temperatures. Bacterial culturability was evaluated over 48 h at 5 °C (**a**) 25 °C (**b**), and 37 °C (**c**). Data are presented as mean CFU/mL $\:\pm\:$ standard error

Potential probiotic *Lactobacillus* strains should ideally possess thermotolerance to different storage temperatures. While the optimum growth temperature for most *Lactobacillus* species is 30–45 °C, storage conditions may vary widely. The thermostability of the 11 *Lactobacillus* species was evaluated at 5, 25 and 37 °C, over 48 h (Fig. 3a, b, c). All *Lactobacillus* species remained culturable at all temperatures over the 48 h experiment (Fig. 3a, b, c). At 5 °C, no significant change in bacterial load was observed. *L. crispatus* and *L. reuteri* exhibited an increase in load but the difference was not significant. At both 25 °C and 37 °C, all strains showed an increase in bacterial titre, indicating that these strains should be stored under refrigeration to maintain a consistent probiotic dose.

### Adhesive capacity of *Lactobacillus* species to segments of ileum and caecum

**Fig. 4:**
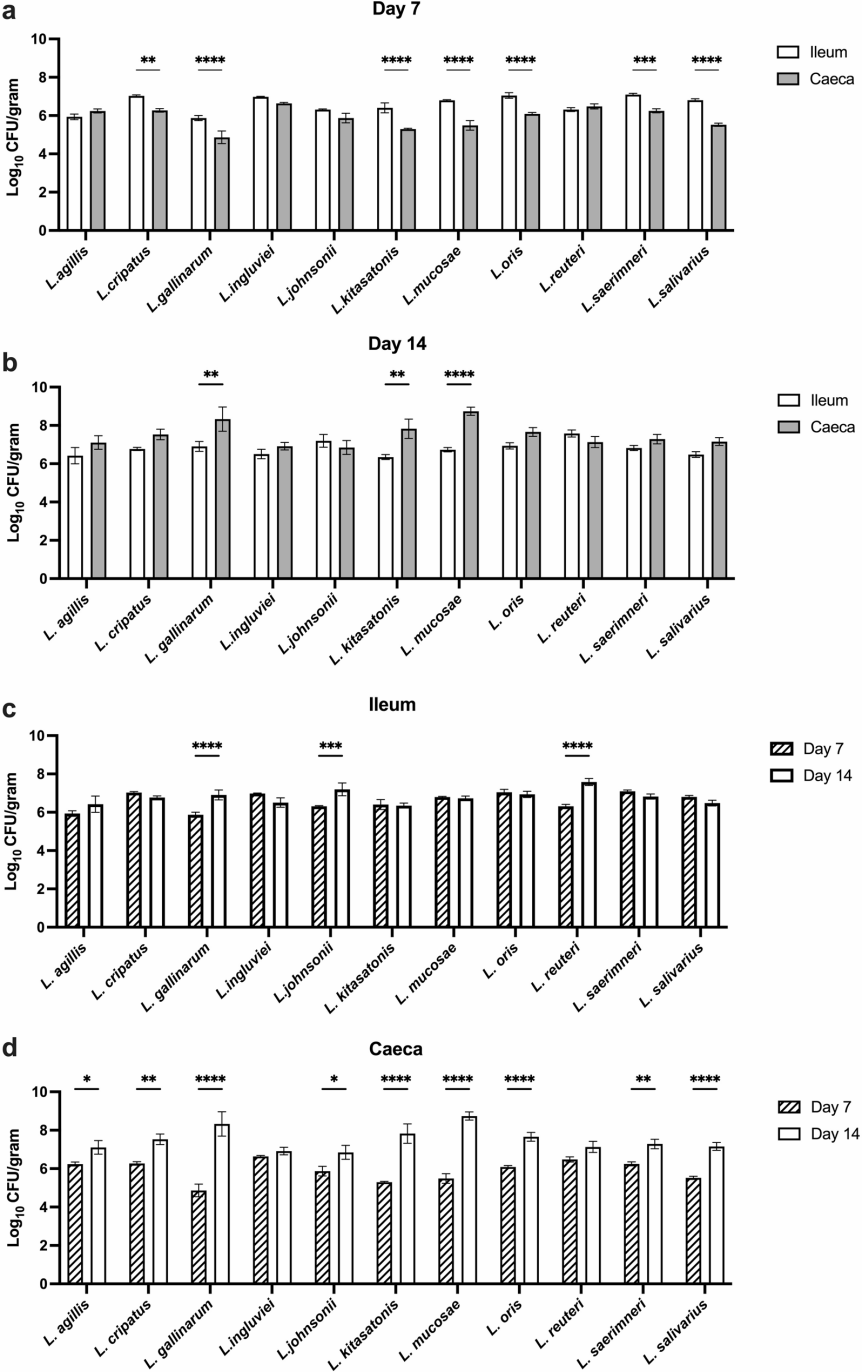
Adhesion of *Lactobacillus* species to the ileal and caecal segments. Ileal and caecal punches were collected from 7 and 14 day old chicks to assess in vitro adhesion. *Lactobacillus* adhesion was compared between ileal and caecal punches at day 7 (**a**) and 14 (**b**). Differences in bacterial adhesion for ileum (**c**) and caeca (**d**) to day 7 and 14 tissue punches. * denotes *p* < 0.05, ** *p* < 0.01, *** *p* < 0.001, and **** *p* < 0.0001

Adhesive capacity reflects the ability of *Lactobacillus* strains to colonise the gastrointestinal tract [31]. Adherence to the intestinal epithelial surface can also help outcompete pathogenic bacteria for attachment sites. In this study, experiments were conducted to evaluate the adhesion capacity to ileal and caecal tissue collected at 7- and 14-days post-hatch (Fig. 4).

All *Lactobacillus* strains demonstrated adhesive properties to 7- and 14-day old sections of both ileal and caecal tissue. *L. crispatus*, *L. gallinarum*, *L. kitasatonis*, *L. mucosae*, *L. oris*, *L. saerimneri*, and *L. salivarius* exhibited significantly higher adhesion to ileal tissue from 7-day old chicks compared to the cecum (p $\:\le\:0.001)$ (Fig. 4a). *L. gallinarum*, *L. kitasatonis*, and *L. mucosae* all exhibited significantly higher adhesion to 14-day old caecal segments (p $\:\le\:0.001)$ (Fig. 4b).

Significant developmental changes occur in the gastrointestinal tract in the first few weeks post-hatch which may contribute to bacterial adhesion and colonisation. Adhesion to ileal and caecal segments was compared between 7- and 14-day old tissues for all *Lactobacillus* species included in this study. Between days 7 and 14, minimal differences were observed in bacterial adhesion to the ileum (Fig. 4c). *L. gallinarum*, *L. johnsonii*, and *L. reuteri* all exhibited significantly higher (p $\:\le\:$ 0.05) adhesive capacity to 14 day old ileal segments (Fig. 4c). For caecal sections, all *Lactobacillus* strains, except *L. ingluvei* and *L. reuteri*, exhibited substantially higher (p $\:\le\:$ 0.05) adhesion to day 14 tissue (Fig. 4d).

### Auto- and co-aggregation properties of *Lactobacillus* spp

**Fig. 5:**
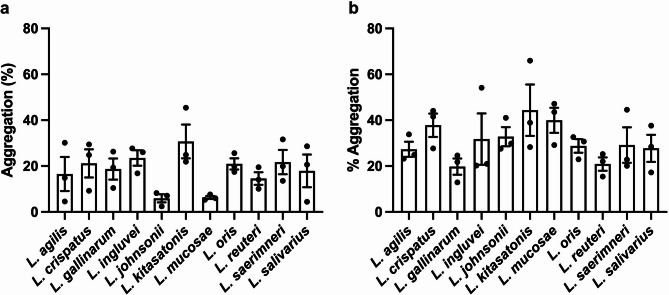
Auto and co-aggregation capacity of *Lactobacillus* strains. All strains exhibited autoaggregation (**a**) not did not differ significantly. All species exhibited co-aggregation (**b**) when co-incubated with *Salmonella* Typhimurium, but no significant difference was observed. Data are presented as the mean % aggregation $\:\pm\:$ standard error

The ability of *Lactobacillus* species to form multicellular aggregates also contributes to colonisation of the gastrointestinal tract [36]. Here, the auto-aggregation capability of the 11 *Lactobacillus* species isolated from layer hen faeces was evaluated. Auto-aggregation did not vary significantly between the tested isolates. *L. kitasatonis* exhibited the highest mean % auto-aggregation of 30.7 ± 7.4%. (Fig. 5a) while *L. johnsonii* and *L. mucosae* had the lowest capacity to auto-aggregate with mean % aggregation of 5.9 $\:\pm\:$ 1.7% and 6.3 $\:\pm\:$ 0.7%, respectively (Fig. 5a). All other *Lactobacillus* isolates exhibited moderate aggregation ranging between 14.6 and 23.5%.

Co-aggregation is the formation of multicellular aggregates between genetically distinct species. This property may contribute to controlling pathogen colonisation of the gut [37]. In the present study, the ability of the *Lactobacillus* isolates to co-aggregate with a layer hen associated *Salmonella* Typhimurium isolate was evaluated. All *Lactobacillus* species exhibited the ability to co-aggregate with *Salmonella* Typhimurium and no significant difference in co-aggregation was observed between isolates (Fig. 5b). *L. kitasatonis* exhibited the highest co-aggregation with *Salmonella* Typhimurium with a mean % aggregation of 44.4 ± 11.2%. The lowest co-aggregation was observed for *L. gallinarum* (19.8 ± 3.5%) and *L. reuteri* (20.0 ± 2.9%). While *L. mucosae* and *L. johnsonii* exhibited weak auto-aggregation, both had strong co-aggregation with *Salmonella* Typhimurium with mean % aggregation of 40.0% ± 5.5% and 32.8% ± 4.15%, respectively.

### *Lactobacillus* co-culture inhibits *Salmonella* Typhimurium

**Fig. 6:**
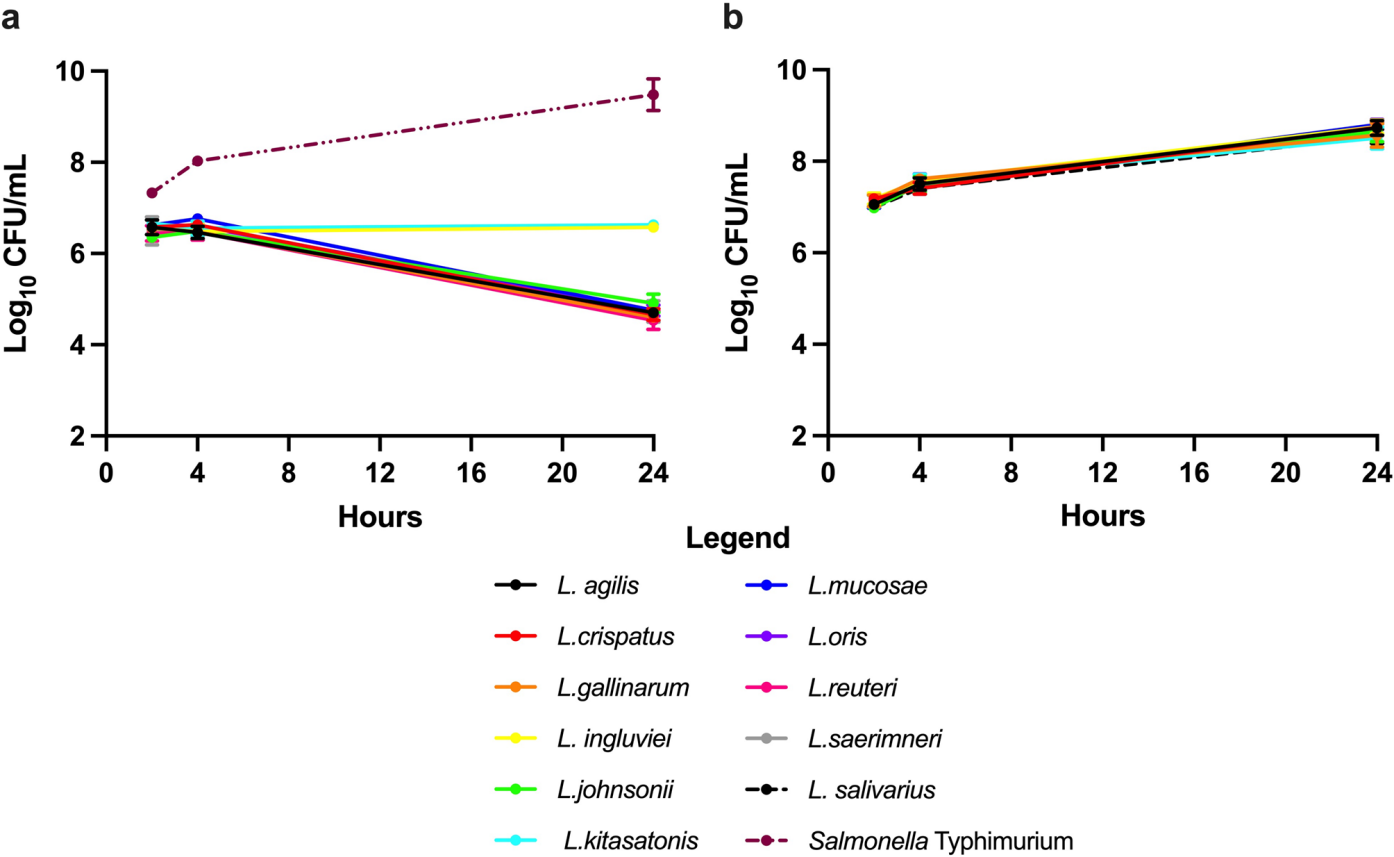
Inhibition of *Salmonella* Typhimurium. The growth of *Salmonella* Typhimurium was significantly inhibited when co-cultured with *Lactobacillus* (**a**). *L. ingluvei* and *L. johnsonii* prevented *Salmonella* growth in the co-culture but a decline in titre was observed for all other *Lactobacillus* species. Growth of *Lactobacillus* species was not negatively impacted by the presence of *Salmonella* (**b**)

The inhibition of *Salmonella* Typhimurium during co-culture with each of the *Lactobacillus* strains was also investigated. After 4 h of incubation, no change in culturable *Salmonella* Typhimurium was observed for any of the treatment groups (Fig. 6a). After 24 h, however, a significant reduction (p $\:\le\:$ 0.05) in *Salmonella* culturability was observed for co-cultures with *L. agilis*, *L. crispatus*, *L. gallinarum*, *L. kitasatonis*, *L. mucosae*, *L. oris*, *L. reuteri*, *L. saerimneri*, and *L. salivarius* (Fig. 6a). No change in the load of *Salmonella* Typhimurium was observed for the *L. ingluveii* and *L. johnsonii* treatment groups. The *Salmonella* Typhimurium control (no *Lactobacillus*) exhibited a 2 log increase, highlighting the impact of *Lactobacillus* co-culture. The load of each *Lactobacillus* strain was also evaluated (Fig. 6b). All species exhibited an increase in bacterial load (p $\:\le\:$ 0.01) indicating that there was no inhibitory effect of *Salmonella* Typhimurium on the *Lactobacillus* species (Fig. 6b).

## Discussion

*Lactobacillus* species have received significant attention as potential probiotics due to their role in promoting a healthy gut microbiota, maintaining the gastrointestinal barrier, inhibiting pathogenic bacteria, and supporting immune development in chickens [38, 39]. Probiotic bacterial strains are selected primarily for their genotypic and phenotypic properties but there is mounting evidence that host-species specificity may also be important. Adaptation to the host can contribute to colonisation of the gut contributing to probiotic efficacy [40]. To date, a large majority of the literature investigating *Lactobacillus* as probiotic strains for poultry have been focussed on broilers.

In the present study, *L. agilis*, *L. crispatus*,* L. gallinarum*,* L. ingluviei*,* L. johnsonii*,* L. kitasatonis*,* L. mucosae*, *L. oris*, *L. reuteri*, *L. saerimneri* and *L. salivarius* were isolated from commercial brown layer hen faeces and evaluated for their phenotypic potential as probiotic bacterial strains. A similar profile of *Lactobacillus* species were reported following culture isolation from the cloaca [20] and faeces [41] of different layer hen breeds. It should be noted that *Lactobacillus mucosae* was first isolated from pig faeces [42] and has subsequently been characterised from human [43], cattle [44], geese [45], and goat milk [46] but to date, has not previously been reported from broiler or layer hen breeds. Diet, production type, and geographical location may all contribute to the profile of *Lactobacillus* strains present in the gut of layer hens. Further studies are necessary to provide a comprehensive understanding of the specific *Lactobacillus* species present in the layer hen gastrointestinal tract.

Probiotic microbes are exposed to a range of stressors during manufacturing, such as fermentation, cultivation, and storage, as well as host related stressors encountered in the gastrointestinal tract [47]. Isolates of the eleven different species were further evaluated for their phenotypic characteristics as potential probiotic strains. Both acid tolerance and resistance to gastric juices are important properties of potential probiotic bacteria, enabling them to survive in the gastrointestinal tract. The pH of the poultry gastrointestinal tract ranges between 2.6 (gizzard) and 6.3 (large intestine) [32]. In the present study, *Lactobacillus* acid tolerance was evaluated at pH 2, 3, and 4 to cover the most acidic environments in the gut. All isolates of *L. agilis*, *L. mucosae*, *L. reuteri*, *L. saerimneri*, and *L. salivarius* exhibited acid tolerance. These results are consistent with previous studies where poultry (broiler or goose) isolates of *L. reuteri* [45, 48], *L. saerimneri* [45, 48], and *L. mucosae* [45] remained culturable over four hours at pH 2. The *L. oris* isolates from the present study were the most acid sensitive which is consistent with strains of *L. oris* strains isolated from geese [45]. Interestingly, all five *L. agilis* strains isolated in the present study were highly acid-tolerant, contrasting with results reported for the same species isolated from geese, which were found to be acid sensitive [45]. Pepsin, a proteolytic enzyme, is secreted in the proventriculus and under acidic conditions is bactericidal [49]. Tolerance to simulated gastric juice at pH 3 was also evaluated and *L. agilis* and *L. ingluvei* isolates exhibited the greatest sensitivity. *L. crispatus* isolates were the most tolerant.

The bile salts possess antimicrobial activities and can inhibit the growth and survival of gut bacteria [50]. The lipophilic nature of bile salts makes them highly toxic to bacterial cells. The bacterial cell membrane is their primary target but once inside the cell, they can acidify the cytoplasm and induce DNA damage (reviewed in [33]). Thus, resistance to bile salts is an important property of probiotic species. In the small intestine of poultry birds, bile salt concentrations can range between 0.2 and 2.0% depending on diet and time of day [51]. In the present study, tolerance to bile salts was evaluated at 1, 3, and 5%. *L. kitasatonis* exhibited the highest bile salt sensitivity while *L. johnsonii* was the most tolerant. All other *Lactobacillus* species exhibited moderate tolerance at 1% but showed increasing sensitivity with increasing concentrations. Through repetitive exposure, such as sub-culture, strains of *Lactobacillus* and *Bifidobacterium* have been shown to adapt to increasing concentrations of bile salts [33]. This adaptation has been shown to confer resistance to other stressors such as pH and oxidative damage [52].

Increasing concentrations of sodium chloride can be used to model bacterial tolerance to osmotic stress, which causes bacterial cells to lose water and reduce production of important metabolites some of which contribute to gut health [53]. *Lactobacillus* strains have inherent tolerance to salt [54]; however, certain strains may be more susceptible than others. Low levels of salt and organic material can negatively affect the lactic acid production of *Lactobacillus* strains [55]. All *Lactobacillus* strains in the present study exhibited tolerance to sodium chloride and exhibited cellular replication event at the highest concentration of 7%. A separate study demonstrated that strains of *L. salivarius*,* L. johnsonii*, and *L. agilis* were non-viable when exposed to 6.5% of NaCl [56], which is inconsistent with data obtained in the present investigation. This could be attributed to strain variation.

It is ideal for probiotic bacteria to remain viable under a range of conditions for as long as possible to ensure that they can colonise the gut and exert a positive effect. Viability can be compromised by storage temperature. Results obtained in the present study have demonstrated that the *Lactobacillus* strains retained culturability at 5 °C, 25 °C, and 37 °C suggesting their ability to withstand varying storage temperatures.

Adhesion of *Lactobacillus* to the epithelium of the gastrointestinal tract is an important attribute that contributes to host colonisation [31]. Additionally, adhesion of pathogens to the gut epithelium is linked with infection control. Probiotic bacterial species with strong adhesion capacity can disrupt the interaction between the host and pathogen, limiting the potential for disease [57]. At day 7, *L. crispatus*, *L. gallinarum*, *L. kitasatonis*, *L. mucosae*, *L. oris*, *L. saerimneri*, and *L. salivarius* all exhibited significantly higher adhesion to ileal tissue compared with the caeca but at day 14, adherence to the caecal tissue was greater. As a chick ages, changes in gut morphology occur. In the caeca, the number of goblet cells increases [58], which is likely coupled with higher rates of mucin production. In the present study, bacterial adhesion to day 14 caecal tissue was substantially higher than tissues collected at day 7. Future experiments are needed to establish the changes in the gastrointestinal epithelium and the relationship with *Lactobacillus* adhesion, which could influence timing of probiotic administration.

Auto-aggregation is an important mechanism by which probiotic bacteria can resist mechanical forces in the gut and colonise the host [31]. In the present study, *L*. *kitasatonis* exhibited the highest auto-aggregation of all *Lactobacillus* species tested. This property likely contributes to bacterial adherence to the gut epithelium, although *L. kitasatonis* did not exhibit significantly higher adherence to intestinal tissue in-vitro. To date, the auto-aggregation activity of *L. kitasatonis* strains has not been well characterised. Previous studies have found that *L. ingluviei*,* L. johnsonii*,* L. agilis*,* L. saerimneri*,* L. mucosae*,* L. gallinarum*, and *L*. *reuteri*, exhibited weak auto-aggregation ability [59–61]. Similar results were observed in the present study suggesting that these species possess a weak ability to form aggregates to colonise in the gut.

Co-aggregation is a parameter used to evaluate the capacity of potential probiotic strains to interact with pathogenic bacteria. This phenomenon underscores the potential of *Lactobacillus* species to effectively combat pathogens through co-aggregation and competitive exclusion. In this study, all *Lactobacillus* species exhibited strong co-aggregation activity with *Salmonella* Typhimurium, suggesting their potential to engage in competitive interactions or inhibit the colonisation of *Salmonella* Typhimurium in the gut of layer hens. Inhibition of pathogens is important benefit of probiotic usage [62]. This effect can be attributed, in part, to the production of SCFAs [63]. The resulting decrease in gut pH, brought about by the release of H^+^ ions, hinders the invasion and colonisation of pathogens. The present study demonstrated that *L. agilis*, *L. gallinarum*, *L. reuteri*, *L. crispatus*, *L. johnsonii*, *L. salivarius*, *L. saerimneri*, *L. oris*, and *L. mucosae* significantly reduced the culturability of *Salmonella* Typhimurium. This is consistent with previous studies, which demonstrated that *L. salivarius* and *L. johnsonii* exhibit competitive exclusion against *Salmonella* Typhimurium in the gastrointestinal tract of layer hens [64]. Further in-vivo experiments are required to characterise the exclusion ability of the other *Lactobacillus* species against other layer hen pathogens.

## Conclusions

In the present study, eleven *Lactobacillus* species were isolated from faeces of commercial brown layer hens. The in-vitro experiments indicate that several of the *Lactobacillus* strains isolated in this study possess probiotic properties. Whole genome sequencing of these isolates is an important next step in *Lactobacillus* characterisation. Identification of deleterious genes such as antimicrobial resistance genes or plasmids, virulence factors, and hazardous metabolic genes such as nitroreductase and biogenic amine production are a crucial aspect of probiotic development. Additionally, in vivo experiments are required to evaluate their capacity to colonise and persist in the host. The potential effects of *Lactobacillus* species as probiotics on gut microbiota and overall health parameters require further in-vivo investigation to determine their suitability. The rarely isolated species *L. mucosae* and *L. kitasatonis* warrant further in-vivo investigation as potential probiotics.

## Data Availability

Data are available from the corresponding author on request.
